# Hyperthyroid Hypokalemic Periodic Paralysis in a Nepali Male; A Case Report

**DOI:** 10.1002/ccr3.71023

**Published:** 2025-09-25

**Authors:** Ashish Tamang, Prakash Sapkota, Sankalpa Humagain, Kemisha Dhakal, Chhavi Sachdeva, Deshant Shah, Prakriti Lamsal

**Affiliations:** ^1^ Department of Research and Development, Dhulikhel Hospital Kathmandu University School of Medical Sciences Dhulikhel Nepal; ^2^ Gastroenterology and Interventional Endoscopy Unit, Department of Internal Medicine Kathmandu University School of Medical Sciences, Dhulikhel Hospital Dhulikhel Nepal; ^3^ Kathmandu University School of Medical Sciences, Dhulikhel Hospital Dhulikhel Nepal

**Keywords:** case report, hyperthyroidism, hypokalemic periodic paralysis, thyrotoxic periodic paralysis

## Abstract

Hyperthyroid Hypokalemic Periodic Paralysis (HHPP), marked by acute weakness and hypokalemia. Prompt potassium replacement and hyperthyroidism management are essential to prevent life‐threatening outcomes. This case highlights its presentation in a Nepali male, reinforcing the need for high clinical suspicion.

## 1 Introduction

Hyperthyroid Hypokalemic Periodic Paralysis (HHPP) is an uncommon but potentially dangerous complication in patients with hyperthyroidism, characterized by acute proximal symmetrical lower limb weakness and hypokalemia, which can extend to all four limbs and respiratory musculature [1]. The hypokalemia associated with this disease is thought to be due to increased activity of the sodium‐potassium adenosine triphosphatase (Na‐K ATPase) pump and mutations in genes encoding inwardly rectifying potassium (Kir) channels in skeletal muscle [2].

HHPP predominantly affects Asian populations, occurring in about 2% of patients with any form of thyrotoxicosis [3]. It is a common complication of hyperthyroidism, particularly in Asian men, but is increasingly observed in Western countries as well [4]. Graves' disease, the leading cause of hyperthyroidism worldwide, is the most frequent underlying cause of HHPP [5].

Misdiagnosis of HHPP is common due to its clinical similarity to familial periodic paralysis. Both conditions present with identical neuromuscular symptoms, making it imperative for physicians to detect subtle signs of hyperthyroidism in patients presenting with hypokalemic periodic paralysis [4].

Effective management of HHPP involves rapid potassium replacement and normalization of thyroid hormones to mitigate this potentially lethal complication [4].

We present a case of HHPP in a young Nepali male with Graves' disease to illustrate the clinical challenges and management strategies.

## 2 Case Description

A 30‐year‐old male presented to the emergency room with complaints of sudden onset bilateral lower limb weakness for 2 h noticed at 5:00 a.m. He reported no pain, bowel or bladder incontinence, upper limb weakness, loss of consciousness, altered behavior, slurring of speech, headache, or facial deviation. There was no history of fever or vertigo. The patient was diagnosed with Graves' disease one month earlier, for which he was on tablet carbimazole 5 mg thrice daily and tablet propranolol 10 mg twice daily. He is vegetarian and does not smoke or consume alcohol.

Upon clinical examination, the patient was alert, and oriented to time, place, and person. His temperature was 36.3°C, respiratory rate was 18 breaths per minute, heart rate was 122 bpm, BP 130/80 mm of Hg and SpO_2_ was 98%. No signs of thyrotoxicosis such as exophthalmos or goiter were noted. Systemic Examination: Respiratory, cardiovascular and abdominal examinations were within normal limit.

CNS: Glasglow Coma Scale: 15/15.

Motor Power: Right Upper Limb: 5/5; Left Upper Limb: 5/5; Right Lower Limb: 4/5; Left Lower Limb: 4/5; Bulk: Normal; Tone: Normal; Reflexes: Present; Sensory: Intact.

## 3 Methods

The initial differential diagnosis included familial hypokalemic periodic paralysis, secondary hypokalemia (e.g., from renal or GI losses), and other neuromuscular disorders like myasthenia gravis.

**FIGURE 1:**
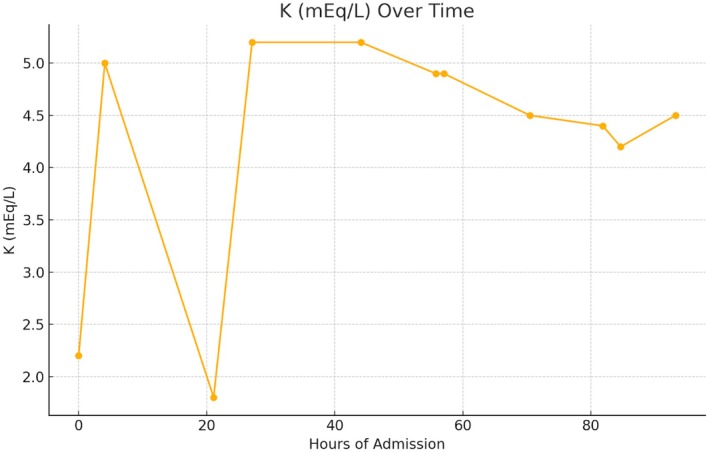
Level of serum potassium over time (hours).

A diagnosis of HHPP was confirmed based on the clinical presentation, laboratory findings of hypokalemia (Serum potassium was critically low at 2.2 mEq/L), and thyroid function tests (TSH levels < 0.01 mIU/L and elevated free T4 levels) indicating hyperthyroidism (Table 1). An EKG was also done which showed sinus tachycardia with no other abnormalities. The patient was immediately treated with intravenous potassium chloride to correct the hypokalemia. Concurrently, Tablet propranolol 10 mg (a beta‐blocker) and Tablet carbimazole 5 mg along with IV fluids were administered to manage the symptoms of hyperthyroidism. The patient was closely monitored for electrolyte levels (Figure 1) and cardiac rhythm. The patient's muscle strength began to improve within hours of potassium replacement, and his potassium levels normalized. He was discharged with a prescription for potassium chloride syrup, Tab propranolol 10 mg BD, and Tab carbimazole 5 mg TDS. The patient was advised to follow a low‐carbohydrate diet to prevent further attacks and scheduled for an endocrinology follow‐up to monitor and manage his thyroid condition.

**TABLE 1:** Laboratory investigations.

Investigations	Patient values	Reference values
Total leukocyte count	7800/μ/L	4000–11,000/μ/L
Hemoglobin	12.5 g/dL	13–17 g/dL
Platelet counts	107 × 10^3^/μ/L	150–450 × 10^3^/μ/L
Serum sodium	136 mEq/L	135–148 mEq/L
Serum potassium	2.2 mEq/L	3.5–5.3 mEq/L
Creatinine	0.5 mg/dL	0.6–1.3 mg/dL
Urea	4 mg/dL	10–45 mg/dL
Serum magnesium	1.8 mg/dL	1.6–2.3 mg/dL
Creatine kinase	119 U/L	38–174 U/L
fT3	8.25 pg/mL	2.77–5.27 pg/mL
fT4	2.89 ng/dL	0.78–2.19 ng/dL
TSH	< 0.01 mIU/L	0.46–4.08 mIU/L
ESR	10 mm/h	< 15 mm/h
Random glucose	110 mg/dL	60–150 mg/dL
Urine routine and microscopy	Nothing abnormal detected (WNL)	—
Serum calcium	8.8 mg/dL	8.4–10.2 mg/dL
Serum potassium (after correction)	4.7 mEq/L	3.5–45.3 mEq/L

The patient's muscle strength began to improve within hours of potassium replacement, and his potassium levels normalized. He was discharged with a prescription for potassium chloride syrup, Tab propranolol 10 mg BD, and Tab carbimazole 5 mg TDS. The patient was advised to follow a low‐carbohydrate diet to prevent further attacks and was scheduled for an endocrinology follow‐up to monitor and manage his thyroid condition.

## 4 Discussion

Hyperthyroid Hypokalemic Periodic Paralysis (HHPP) is a rare channelopathy manifesting as abrupt attacks of muscular weakness concurrent with hypokalemia in thyrotoxic patients [6]. Epidemiologically, this condition demonstrates a striking predilection for individuals of Asian descent, with an incidence of approximately 2% among those with hyperthyroidism, typically presenting between the ages of 20 and 40 [7, 8]. This contrasts with non‐Asian populations, which are estimated to be between 0.1% and 0.2% [3, 7].

Although the real mechanism behind the cause of periodic paralysis is unclear, it is thought the pathophysiology involves an increase in the activity of the sodium‐potassium‐adenosine triphosphate (Na/K‐ATPase) pump in skeletal muscle cells, driven by elevated thyroid hormones [4]. This leads to an intracellular shift of potassium, causing hypokalemia and subsequent muscle weakness [2]. Additionally, genetic factors, such as mutations in the genes encoding inwardly rectifying potassium (Kir) channels in skeletal muscle, may predispose individuals to HHPP by further enhancing potassium influx into cells. Although familial forms of the condition exist and are usually autosomal dominant, most cases, like ours, are sporadic [6]. The higher prevalence in Asian populations may be attributed to genetic and environmental factors unique to this demographic. However, the increasing recognition of HHPP in Western countries suggests that it is a global issue, likely underdiagnosed outside of Asia [4].

The classic clinical picture involves acute, flaccid, and symmetrical weakness that primarily targets the proximal muscle groups of the lower limbs. The potential for progression to involve all extremities and even respiratory muscles constitutes a medical emergency [4]. Thus, a high index of suspicion for hyperthyroidism is essential when diagnosing HHPP in patients presenting with hypokalemic periodic paralysis [4].

The condition is precipitated by a combination of thyrotoxicosis, environmental factors such as a high carbohydrate load or intense exercise, and an underlying genetic mutation.

A retrospective study done by Ko et al. reported that 75% of TPP attacks occurred between 9:00 p.m. and 9:00 a.m., with peaks between 9:00 p.m.–12 Midnight and 3:00 a.m.–9:00 a.m. The evening peak may correlate with rest after exercise or meals, while the early morning peak might reflect metabolic or hormonal fluctuations during sleep [9]. These findings highlight the role of precipitating factors such as carbohydrate‐rich meals and circadian influences in TPP pathogenesis [9]. Similarly, in our case, the attack was noted at 5 a.m. While our patient had no family history of HHPP, familial forms are autosomal dominant and usually present in early teenage years. For instance, a case involving a 14‐year‐old African American male diagnosed with this disease highlights the hereditary nature of the condition [10].

A comparable case has been reported wherein the patient felt weakness after waking up in the morning, where he could not move both of his lower limbs, with no respiratory distress, palpitations, blurred vision, slurred speech, or bowel and bladder incontinence [11].

The differential diagnosis of HHPP includes conditions such as normokalemic and hyperkalemic periodic paralysis, Andersen‐Tawil syndrome, secondary hypokalemia, myasthenia gravis, and paramyotonia congenita [12]. Distinguishing HHPP from these disorders relies on differences in serum potassium levels during attacks, genetic testing, and clinical features. For instance, normokalemic and hyperkalemic periodic paralysis typically present with normal or elevated serum potassium levels during episodes, whereas HHPP is marked by hypokalemia. Andersen‐Tawil syndrome involves periodic paralysis with characteristic facial and skeletal anomalies and cardiac manifestations due to mutations in the KCNJ2 gene [6]. Paramyotonia congenita, caused by SCN4A gene mutations, features myotonia triggered by cold and exercise, without potassium level changes. Secondary hypokalemia results from underlying systemic conditions affecting renal or endocrine systems, and myasthenia gravis presents with predictable muscle weakness triggered by exertion, distinguishing it from the episodic weakness in HHPP. Understanding these differences is crucial for accurate diagnosis and effective management [6].

Preventive measures include dietary modifications, particularly reducing carbohydrate intake, as carbohydrate‐rich meals can precipitate attacks by further stimulating insulin secretion and Na/K‐ATPase activity. Ongoing management of hyperthyroidism is crucial to prevent recurrence of HHPP episodes [6].

Prophylactic potassium supplementation between attacks has not proven to be effective and is not generally recommended [1]. Non‐selective beta‐blockers, such as propranolol, can be beneficial in alleviating neuromuscular symptoms by reducing the intracellular shift of potassium and phosphate. In cases unresponsive to potassium replacement, intravenous propranolol (1 mg every 10 min up to three doses) may be administered [13]. It is also crucial to monitor and avoid medications that induce hypokalemia, such as glucocorticoids, in patients with HHPP. The definitive treatment involves reducing thyroid hormone levels and achieving a euthyroid state. Depending on the underlying cause of hyperthyroidism, treatment may involve antithyroid drugs, radioactive iodine, or surgery. Additionally, patients should avoid precipitating factors such as strenuous exercise and high‐carbohydrate meals to prevent recurrence of HHPP episodes [14, 15].

Prompt identification and correction of hypokalemia is crucial to reverse motor weakness and prevent respiratory compromise and fatal cardiac arrhythmias [6, 16]. In a similar case, acute treatment included 40 meq of oral potassium, 1 L of saline with 20 meq potassium, and magnesium supplementation. For recurrent episodes, the patient was managed with 20 mg methimazole twice daily and 20 mg propranolol three times daily. In our case, 5 mg carbimazole thrice daily and 10 mg propranolol twice daily were effective in preventing further episodes, emphasizing the importance of individualized long‐term hyperthyroidism management [10].

## 5 Conclusion

This case highlights the critical importance of recognizing HHPP as a differential diagnosis in patients presenting with acute muscle weakness, particularly in those with a known history of hyperthyroidism. Early diagnosis and prompt treatment are vital to avoid severe complications and ensure patient recovery. Clinicians should maintain a high index of suspicion for HHPP in hyperthyroid patients with hypokalemic paralysis and implement appropriate therapeutic interventions without delay in order to prevent the incidence of severe complications, ensuring better patient outcomes.

## Author Contributions

**Ashish Tamang:** conceptualization, data curation, investigation, methodology, project administration, resources, supervision, validation, visualization, writing – original draft, writing – review and editing. **Prakash Sapkota:** conceptualization, data curation, formal analysis, investigation, methodology, project administration, resources, supervision, validation, visualization, writing – original draft, writing – review and editing. **Sankalpa Humagain:** formal analysis, investigation, methodology, project administration, supervision, visualization, writing – original draft, writing – review and editing. **Chhavi Sachdeva:** writing – original draft, writing – review and editing. **Deshant Shah:** writing – original draft, writing – review and editing. **Kemisha Dhakal:** writing – original draft, writing – review and editing. **Prakriti Lamsal:** writing – original draft, writing – review and editing.

## Ethics Statement

Ethical approval was not required for this study by local or national guidelines. Written informed consent was obtained from the patient for publication of details of their medical care.

## Consent

Written informed consent was obtained from the patient for the publication of this report following the journal's patient consent policy.

## Conflicts of Interest

The authors declare no conflicts of interest.

## Data Availability

The authors of this manuscript are prepared to provide Supporting Information concerning the case report upon official request. All data generated or analyzed during this study is included in this article. Further inquiries can be directed to the corresponding authors.
